# The Study of Amidoxime-Functionalized Cellulose Separate Th(IV) from Aqueous Solution

**DOI:** 10.3390/gels8060378

**Published:** 2022-06-15

**Authors:** Yiling Zhi, Guojian Duan, Zhiwei Lei, Hui Chen, Haobo Zhang, Huining Tian, Tonghuan Liu

**Affiliations:** 1 Northwest Collaborative Innovation Center for Traditional Chinese Medicine Co-Constructed by Gansu Province & MOE of PRC, College of Pharmacy, Gansu University of Chinese Medicine, Lanzhou 730000, China; zhiyiling1999@126.com (Y.Z.); zhhb@gszy.edu.cn (H.Z.); tian05082018@126.com (H.T.); 2 Radiochemistry Laboratory, School of Nuclear Science and Technology, Lanzhou University, Lanzhou 730000, China; leizhw17@lzu.edu.cn (Z.L.); liuth@lzu.edu.cn (T.L.); 3 Key Laboratory of Special Function Material and Structure Design Dinistry Education, School of Nuclear Science and Technology, Lanzhou University, Lanzhou 730000, China

**Keywords:** adsorption, amidoxime, cellulose, Th(IV)

## Abstract

Selective extraction of low-concentration thorium (Th(IV)) from wastewater is a very important research topic. In this paper, amidoxime cellulose was synthesized, and its composition and structure were characterized by FT-IR, SEM, XPS, and elemental analysis. The adsorption experiment results showed that the adsorption reaction was a spontaneous exothermic process. When the solid–liquid ratio was 0.12 g/L and the pH value was 3.5, the adsorption percentage of the Th(IV) in water onto amidoxime-functionalized cellulose (AO-CELL) could reach over 80%. The maximum adsorption capacity can reach to 450 mg/g. At the same time, the adsorption selectivity, desorption process and reusability of the material were also studied. The results showed that the AO-CELL had a good selectivity for Th(IV) in the system with Sr^2+^, Cu^2+^, Mg^2+^, Zn^2+^, Pb^2+^, Ni^2+^, and Co^2+^ as co-ions. In the nitric acid concentration of 0.06 mol/L system, the AO-CELL desorption rate of Th(IV) can reach 95%, and the adsorption rate of Th(IV) in aqueous solution of AO-CELL is still above 60% when the AO-CELL is reused four times. The above results show that the amidoxime cellulose adsorption material synthesized by our research group has good selective adsorption performance for Th(IV) of a low concentration in an aqueous solution and has a good practical application value.

## 1. Introduction

Thorium is a natural radioactive metal element, and after being bombarded by neutrons, uranium-233 can be obtained, which is abundant in the earth’s crust [1]. Therefore, thorium may be the next generation of nuclear energy material. At the same time, some actinides, such as Np(IV), U(IV), and Pu(IV), have very unstable tetravalent forms, so tetravalent thorium ions are often studied as their analogues [2]. However, Th(IV) is radioactive, carcinogenic, and toxic. Thorium ore, as an important industrial raw material in the nuclear power industry, will inevitably produce thorium-containing wastewater in the process of mining and utilization. When the concentration of Th(IV) reaches a certain value, it will cause irreversible damage to the environment and ecosystem [3]. Thus, it is of great significance to find an economical and efficient method to separate Th(IV) from aqueous solution. Existing techniques for the removal of radionuclides from aqueous environments include chemical precipitation, ion exchange processes, electrolysis methods, adsorption, membrane, and reverse osmosis processes, etc. [4,5,6]. The adsorption method has the advantages of low cost, high efficiency, and simple operation, and has a good practical application value. An excellent adsorbent should have high adsorption capacity and high regeneration capacity, such as polymer/metal oxide, nano oxide, molecular sieve, activated carbon, lignin, clay, polymer, and biopolymer materials [7,8,9,10,11,12,13,14,15], which have been widely studied for removing metal ions or radioactive elements in water systems. For example, Mu. Naushad’s group synthesized novel MOFs material, and used carboxyl and amide groups to separate Th(IV) from an aqueous environment, with an adsorption capacity of 285.7 mg/g [7]. Eman Kamal’s group used a GO/TiO_2_ nanocomposite to capture Th(IV) from aqueous solutions, and the experiment results showed the highest Th(IV) adsorption of 292.32 mg/g at pH 2.5 and 25 °C [16]. Zhu’s group used the reusable porous Al2O_3_-SiO_2_ composites to remove Th(IV) from aqueous solution, with the maximum adsorption capacity reaching 361.60 mg/g and with an adsorption percentage as high as 99.9% in a low initial concentration of the Th(IV) solution [17]. Although there are some good results from such studies at this stage, the demand for adsorption materials with better performance, higher efficiency, and lower cost is still increasing.

Cellulose is the most abundant biological resource in nature. It has the advantages of stable composition, biocompatibility, biodegradation, short regeneration time, wide source, and low cost [18]. There are three hydroxyl groups on each structural unit of cellulose, of which the hydroxyl group on carbon 6 is the most active [19]. In addition to cellulose itself, it can be used as an adsorbent, and the introduction of specific functional groups to the molecular chain of cellulose can realize the development of new uses on the basis of retaining its original properties. The chemical pretreatment methods include TEMPO oxidation, sulfonation, phosphorylation, esterification, cationization, amination, etc. [20,21,22,23], which can not only increase the adsorption sites of cellulose, but also change the morphology of adsorption materials, thus enhancing the interaction with metal ions [24]. Among the numerous coordination functional groups, the amidoxime group could combine with many kinds of metal ions under acidic conditions to form stable chelates. In particular, the use of amidoxime compound to extract uranyl ions from seawater has been widely used [25]. At the same time, the amidoxime group also shows good adsorption capacity for many heavy metals or transition metals ions [26,27].

In this paper, cellulose is used as a stable and easily functionalized platform. Through a simple two-step synthesis, amidoxime cellulose adsorption material is prepared and applied to enrich and separate Th(IV) from aqueous solution. Through the characterization and testing of the adsorption properties of the adsorbent, it is proved that the material not only has stable chemical properties, but also has a good selective adsorption of thorium ions in aqueous solution. A series of factors affecting thorium ion adsorption were studied in detail, including the solid–liquid ratio, the pH value, the initial concentration, the kinetics, and the thermodynamic laws of the adsorption process, while the selectivity and reusability of the adsorption were also tested. The experimental results show that the amidoxime group also has a good adsorption performance for thorium ion, which may be due to the synergistic effect of hydroxyl in cellulose units, which increase the application potential of this type of adsorbent.

## 2. Results and Discussion

### 2.1. Synthesis and Characterization

#### 2.1.1. Synthesis of Nitrile Cellulose (AN-CELL) and Amidoxime Cellulose (AO-CELL)

Cellulose powder (3.00 g) and acrylonitrile (72.00 mL) were added to a dry three-necked, round-bottomed flask (250.00 mL) and stirred under nitrogen protection at room temperature until the mixture was uniform. Tetramethylammonium chloride (0.10 g) deionized aqueous solution (2.00 mL) and sodium hydroxide (1.13 g) deionized aqueous solution (2.00 mL) were successively added to the reaction system. The reaction system was heated to reflux and stirred for 3 h. At the end of the reaction, a certain amount of isopropyl alcohol and glacial acetic acid (1.00 mL) were added to the system to remove excess acrylonitrile and NaOH. This was followed by vacuum distillation to remove most of the solvents, wherein the remaining part was filtered, and the solid part was washed with deionized water (10 mL × 3 times), followed by being vacuum freeze-dried to obtain nitrile cellulose (AN-CELL).

The AN-CELL (1.00 g) and hydroxylamine hydrochloride (7.00 g) were dissolved in methanol (50 mL), and the pH value of the reaction system was adjusted within the range of 5–6 with a methanol–aqueous solution of sodium carbonate (V_MeOH_:V_deionized water_ = 1:1). Then, the reaction system was heated at reflux for 6 h in a nitrogen atmosphere. Finally, the reaction system was cooled to room temperature, the product was suction filtered, and the solid part was washed with deionized water (10 mL × 3 times). The desired product of AO-CELL was obtained by vacuum freeze-drying for 24 h [28,29,30].

**Scheme 1:**
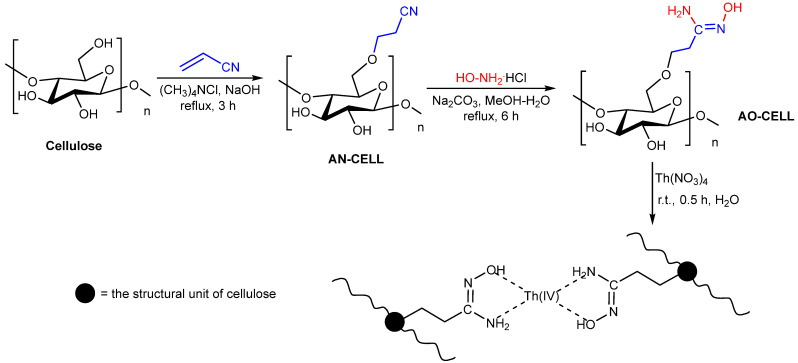
Synthetic reaction equation for AO-CELL and its Th(IV) complexes.

The adsorbent and the prepared Th(IV) raw solution were mixed in a certain proportion in the reaction tube, and the corresponding volume of the nitric acid solution was added to adjust the pH value of the reaction system. Finally, the reaction system was fixed to 5 mL with deionized water, and the reaction tube was sealed. The reaction was performed on a constant temperature shaker for 0.5 h. The synthesis process is shown in Scheme 1.

#### 2.1.2. Elemental Analysis

Table 1 shows the elemental analysis results of cellulose and its derivatives. The results showed that the N content of AN-CELL increased from 0% to 5.89%, indicating that most of the glucose units in the cellulose have undergone the cyanidation reaction, and the content of C, H, and O had changed correspondingly. When cellulose cyanide is further prepared to amidoxime cellulose, the N content is further increased to 10.04%, while the C content is reduced from 49.68% to 44.16%, and the H and O contents have corresponding changes. In the case of subtracting trace water in the material, according to the experimental results and the theoretical calculation, the conversion rate of the cyanidation reaction is about 89%, and the conversion rate of the amidoxime reaction is about 80%.

#### 2.1.3. Spectra of X-ray Photoelectron Spectroscopy

**Figure 1:**
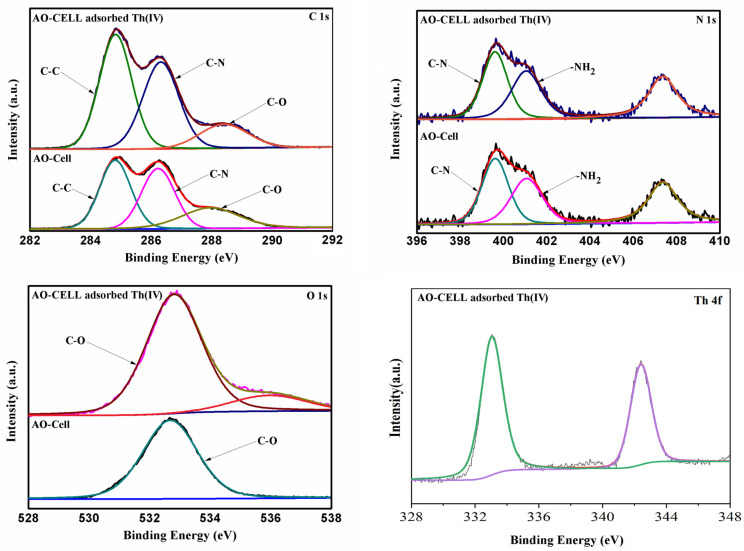
XPS of AO-CELL and its Th(IV) complexes.

The spectra of X-ray photoelectron spectroscopy analysis for AO-CELL and Th(IV)-AO-CELL are shown in Figure 1, and all the tests in this figure were measured at the same conditions. The characteristic peak of thorium appeared after the Th(IV) was adsorbed on AO-CELL, which proves that Th(IV) was adsorbed on the AO-CELL material. In the spectrum of AO-CELL C 1s, there are three different peaks of element C at 284.8, 286.3, and 288.0 eV, corresponding to C-C, C-N and C-O groups, respectively. When the AO-CELL adsorbed the Th(IV), the peak areas of the C-C and C-N groups increased, and the binding energy of the C-O groups increased. In the N 1s spectrum, the N element of AO-CELL can be deconvolved into three peaks at 399.6, 401.1, and 407 eV, corresponding to C-N, -NH_2_, and -NO_3_^−^ [30,31], respectively, but in the Th(IV) complexes, the N 1s spectrum did not change significantly. In the O 1s spectrum, the peak of the O element of AO-CELL appears at 532.7 eV, corresponding to the C-O group [32], but in the Th(IV) complexes, the binding energy increased to 532.9 eV, indicating the electron transfer between Th(IV) and the oxygen atoms in the material [33]. When a new coordination is formed between the O element and Th(IV), the electrons of the O element transfer from its electron-rich orbital to the empty orbital of thorium. The electron excitation of the O element requires X-rays with higher energy, so the binding energy of O 1s in the spectrum becomes higher [34]. The peak area increases, which can be explained by the coordination between C-O and radionuclides during adsorption [35]. It can be observed from Figure 1 Th 4f that peaks belonging to Th 4f_7/2_ and Th 4f_5/2_ orbits appear at 333.1 eV and 342.4 eV, which indicates that thorium ions already exist on the surface of the adsorbent.

#### 2.1.4. Scanning Electron Microscopy and Energy Dispersive Spectroscopy Mapping

**Figure 2:**
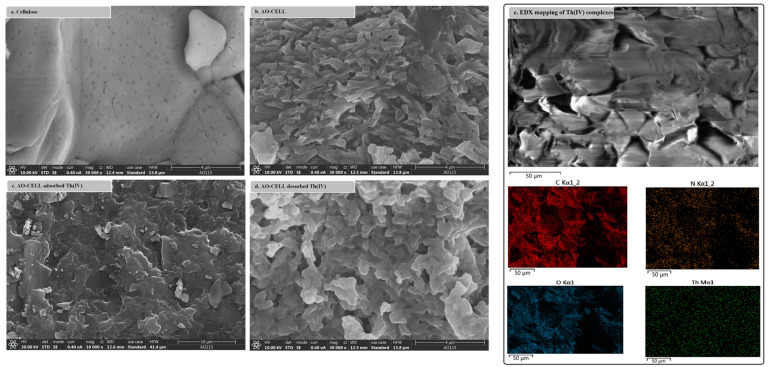
SEM of cellulose its derivatives. (**a**) Cellulose; (**b**) AO-CELL; (**c**) AO-CELL adsorbed Th(IV); (**d**) AO-CELL desorbed Th(IV); (**e**) EDS mapping of Th(IV) complexes.

The morphology of cellulose and its derivative samples were characterized by scanning electron microscopy (SEM), and the results are shown in Figure 2a–d. The SEM results indicated that the microstructure of cellulose is smooth and intact. However, numerous pores exist on the surface of the AO-CELL, so its specific surface area has a significant increase which is conducive to the contact and the interaction between Th(IV) and the functional groups of the AO-CELL. When the AO-CELL adsorbs Th(IV) and reaches the maximum adsorption capacity, its surface presents a smooth and flaky morphology. However, when the Th(IV) ions are removed from the AO-CELL system, the material is restored to its original form as a fluffy and porous state. The above results provide that this kind of adsorption material has an obvious adsorption ability with respect to Th(IV) ions and provides the possibility for the reuse of this material. The appearance of thorium elements in the energy dispersive spectroscopy (EDS) mapping (Figure 2e) indicates that the AO-CELL has a stable adsorption capacity for thorium ions, which reconfirms the conclusion of the SEM images.

#### 2.1.5. Fourier Transform Infrared Spectroscopy

**Figure 3:**
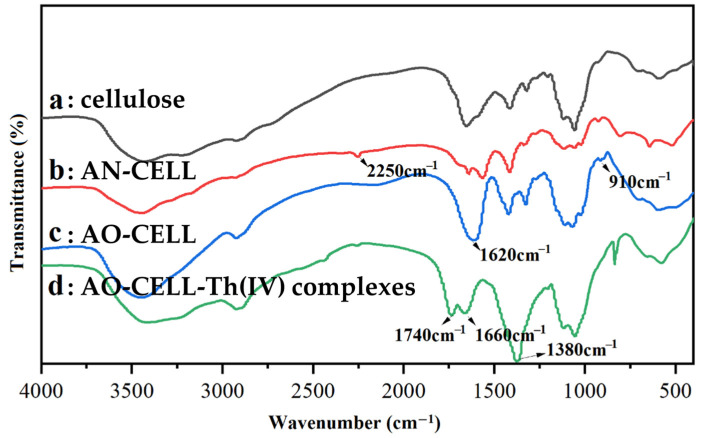
FT-IR spectra of cellulose and its derivatives.

From the Fourier transform infrared spectroscopies (FTIR) of the cellulose and its derivatives (Figure 3), it can be seen that the stretching vibration peak (2250 cm^−1^) of the cyano group C≡N appears in line b in Figure 3, indicating that the cyano group has been successfully introduced into the cellulose molecule [36]. In the infrared spectrum of amidoxime cellulose (line c in Figure 3), the absorption peaks of 1620 cm^−1^ and 910 cm^−1^ appear, which can belong to the stretching vibration peaks of C=N and N-O of the amidoxime group, respectively, and the vibration absorption peak of the cyano C≡N in the AN-CELL has basically disappeared. The above results indicate that part of the cyanide groups have been successfully transformed into amidoxime groups [37,38]. When the amidoxime cellulose is combined with Th(IV) (line d in Figure 3), the C=N characteristic peak at 1620 cm^−1^ splits into two absorption peaks at 1660 cm^−1^ and 1740 cm^−1^, respectively. Meanwhile, a characteristic peak of the C-O-Th stretching vibration characteristic peak was found at 1380 (cm^−1^). The reason for the above results is that the amidoxime group combines with Th(IV) to form a relatively stable five-membered ring structure. Due to the conjugation effect, the vibration absorption peak of C-O weakens and coincides with that of methylene [39,40]. It can be seen from the above results that the AO-CELL has an obvious adsorption effect on Th(IV).

### 2.2. Adsorption Performance Study

#### 2.2.1. Adsorption Kinetics

**Figure 4:**
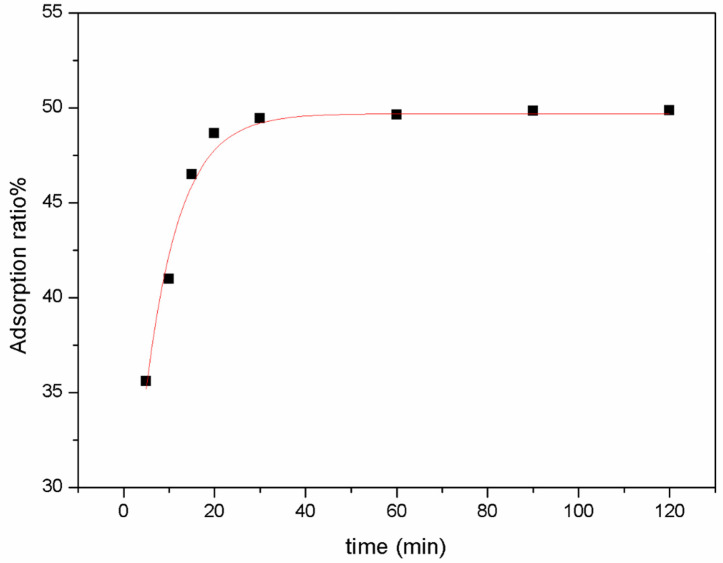
Effect of contact time (solid/liquid ratio = 0.032 g/L; C_0_[Th(IV)] = 19.00 ± 0.10 mg/L; pH = 3.00 ± 0.05; T = 298.15 ± 1.00 K).

Adsorption kinetics studies can predict the rate of the adsorbent and can separate the metal ions from aqueous solution, which is very important for evaluating the efficiency of an adsorbent [41]. Figure 4 shows the variation of the adsorption rate of Th(IV) onto the AO-CELL with the contact time. The results show that the adsorption process can be divided into three stages: in the first stage, there are a large number of empty binding sites on the surface of the AO-CELL, so the adsorption capacity and the adsorption rate at this stage increase rapidly. In the second stage, with the decrease of the surface-active sites of the AO-CELL, the gap between the adsorption and desorption rates decreases, and the increase of the adsorption capacity and the adsorption rate slows down, reaching the maximum value at around 30 min. In the third stage, the adsorption reaction maintains dynamic equilibrium, and the adsorption capacity and the adsorption rate will not change significantly.

The kinetic study of this adsorption reaction is very important to understand the physical and chemistry changes of this process. In this paper, typical adsorption kinetic models are used to fit adsorption data: pseudo-first order and pseudo-second order models, and their linear mathematical expressions, are shown in Equations (1) and (2), respectively:

ln(q_e1_ − q_t_) = lnq_e1_ − K_1_t (1)

t/q_t_ = 1/K_2_q^2^_e2_ + t/q_e2_ (2)

where K_1_ (min^−1^) and K_2_ (g·mg^−1^·min^−1^) are, respectively, the rate constants of the three models, q_e1_ (mg·g^−1^) and q_e2_ (mg·g^−1^) are the calculated equilibrium adsorption capacities of metal ions, while q_t_ (mg·g^−1^) is the adsorption capacity of metal ion at time t.

The results of the kinetic parameters are shown in Table 2. The results show that the dynamic model of the pseudo-second order correlation coefficient, rather than the pseudo-first order kinetics model and the intraparticle diffusion model correlation coefficient, is closer to 1, and at the same time, the pseudo second-order linear mathematical expression derived by the equilibrium adsorption quantity is more consistent with the experimental data. Therefore, the proposed secondary dynamics model can better describe the adsorption reaction process and the adsorption process for chemical adsorption, such that the rates rely more on the availability of the active sites than they do the concentration of metal ions, and have a valence force by sharing or exchanging electrons such as surface complexation [42,43].

#### 2.2.2. Effect of Ionic Strength (Anion/Cation with Different Valence States)

**Figure 5:**
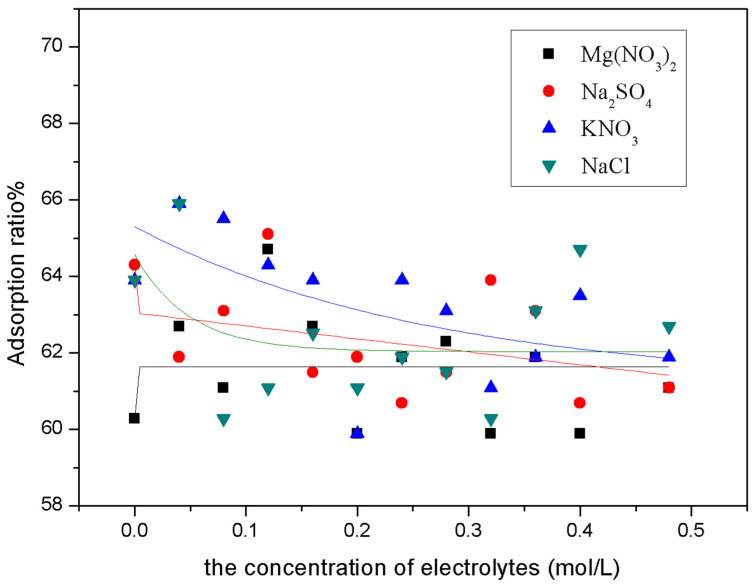
Effect of ionic strength (anion/cation with different valence states). (Experiment conditions: solid/liquid ratio = 0.038 g/L; C_electrolyte_ = 0~0.48 mol/L; C_0_[Th(IV)] = 19.00 ± 0.10 mg/L; pH = 3.00 ± 0.05; T = 298.15 ± 1.00 K; t = 0.5 h).

Generally, there are many kinds of ions in the waste liquid, and the different ionic atmospheres correspond to the different ionic strengths of the solution, which will affect the activity of the Th(IV) and the surface chemistry of the adsorbed materials. In order to investigate the adsorption behavior of Th(IV) on the AO-CELL more comprehensively, Figure 5 shows the influence of different ionic strengths (including different valence states and different concentrations of negative/cation) on the adsorption result of Th(IV) on the AO-CELL. As can be seen from the Figure 5, the solution’s ionic strength and ion-charged species (Na^+^, K^+^, Mg^2+^, NO_3_^−^, Cl^−^, SO_4_^2−^) have little influence on the separation of Th(IV) by the AO-CELL. These results provide data support for the enrichment and separation of thorium ions in mixed systems such as seawater or industrial wastewater [44,45]

#### 2.2.3. Effect of Solid/Liquid Ratio

**Figure 6:**
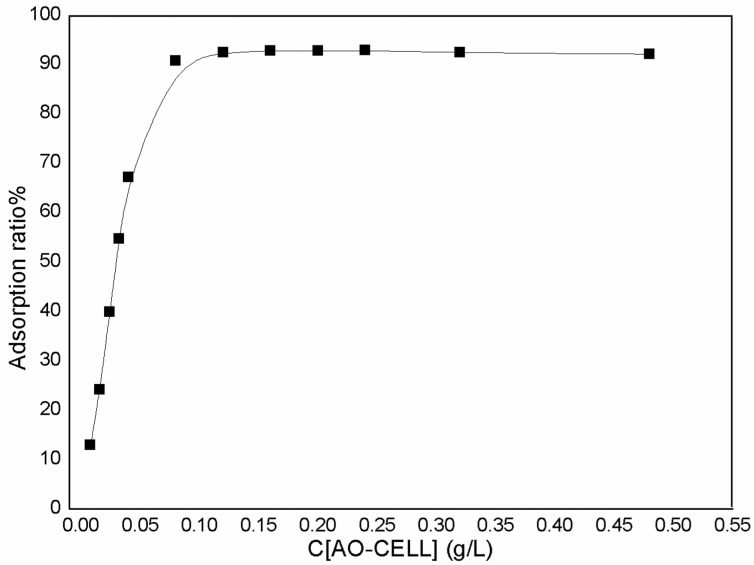
Effect of adsorbent dose on Th(IV) adsorption by CO-CELL. (Experiment conditions: C_0_[Th(IV)] = 19.00 ± 0.10 mg/L; pH = 3.00 ± 0.05; T = 298.15 ± 1.00 K; t = 0.5 h).

The optimal amount of adsorbent was determined by adjusting the solid/liquid ratio, while the other experimental parameters (adjusting the pH value to 3.0, metal ion concentration, contact time, and volume of adsorption medium) were kept constant. As shown in Figure 6, the adsorption percentage (R%) increased significantly with the increase of the amount of adsorbent in the initial stage. However, when the solid/liquid ratio increased to 0.12 g/L, R% remained constant. This is because, at the initial stage, the adsorbent content in the solution is low, providing few adsorption sites. Therefore, even if the adsorbent reaches adsorption saturation, the R% is low, so at this stage, with the increase of adsorption dose, the R% will increased. However, because of the dynamic balance between the Th(IV) trapped by the adsorbents and in the solution, R% will be maintained in a fixed range when the adsorption process reaches equilibrium and does not increase with increasing adsorbed dose. The results also show that at equilibrium, R% is higher than 90%, which indicates that the adsorbent has an excellent adsorption performance for Th(IV).

#### 2.2.4. Effect of pH Value

The pH value of the solution will directly affect the chemical properties of the adsorption material, and the valence or existence state of metal ions, etc., so the pH value of the solution is an important factor that affects the combination process of Th(IV) with the AO-CELL in aqueous solution. Under strong acidic conditions, the stability of the AO-CELL decreases, while under a high pH value, the Th(IV) will undergo a hydrolysis reaction and form the hydroxide precipitation of thorium. Therefore, the pH value of the solution in this part is controlled between 1.5 and 5.5.

**Figure 7:**
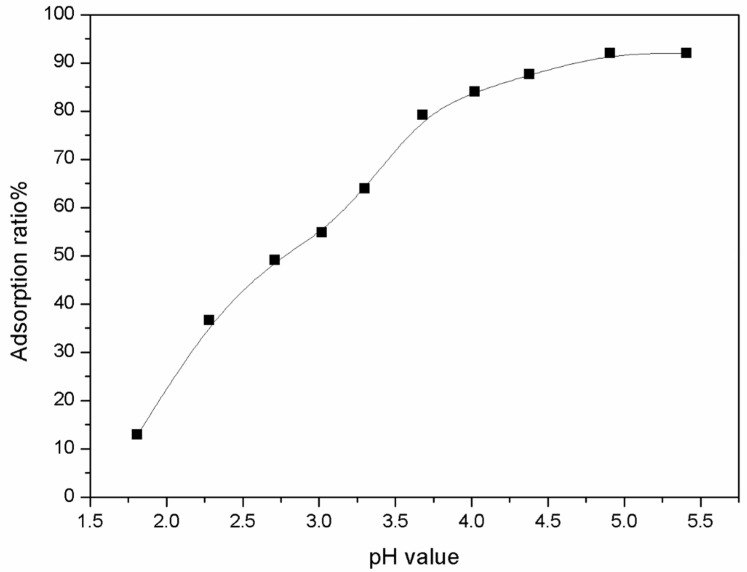
Effect of pH value. (Experiment conditions: C_0_[Th(IV)] = 19.00 ± 0.10 mg/L; solid/liquid ratio = 0.032 g/L; T = 298.15 ± 1.00 K; t = 0.5 h).

As can be seen from Figure 7, with the pH value in the range of 1.5–5.5, the adsorption ratio% increases with the increasing of the solution’s pH value. This is because, at the high acidity conditions, the adsorption groups in the molecular structure of amidoxime cellulose, including the hydroxyl and amidoxime groups, keep their original form or acid salt form, and this is not conducive to the adsorption reaction of Th^4+^. From the perspective of the electronic effect, the relative acidity of the adsorbent increases with the increase of the pH value, and its swelling capacity also increases accordingly. Therefore, it is more conducive to the binding of Th^4+^ with the active site of the adsorption material. Based on the above reasons, the adsorption ratio% of Th(IV) onto the AO-CELL increases with the increasing of the pH value. As is well known, when the pH value of the solution system is higher than 3.0, Th^4+^ begins to undergo an obvious hydrolysis reaction, and a portion of the thorium ions begin as Th(OH)^3+^ and Th(OH)_2_^2+^. When the pH value of the solution system is greater than 3.5, the precipitate of Th(OH)_4_ begins to appear [46]. In order to ensure a more realistic experimental result of the binding rule between the thorium ion and the adsorbent, except for the study of the influence of the pH value on the adsorption process, all the experiments were carried out under the condition of pH = 3.0.

#### 2.2.5. The Selective Adsorption of Th(IV) Ions

There are many kinds of metal ions in the waste liquid system to be treated, so it is important to study the specific adsorption of the target metal ions by the adsorption materials. In this experiment, common metal ions (Sr^2+^, Cu^2+^, Mg^2+^, Zn^2+^, Pb^2+^, Ni^2+^, Co^2+^) in radioactive waste liquid were selected as coexisting competitive ions, and the concentration of all the metal ions were 8.0 × 10^−5^ mol/L. The changes in the concentration of each metal ion were determined by atomic absorption spectroscopy (AAnalyst 700, PerkinElmer Company, Waltham, MA, USA).

**Figure 8:**
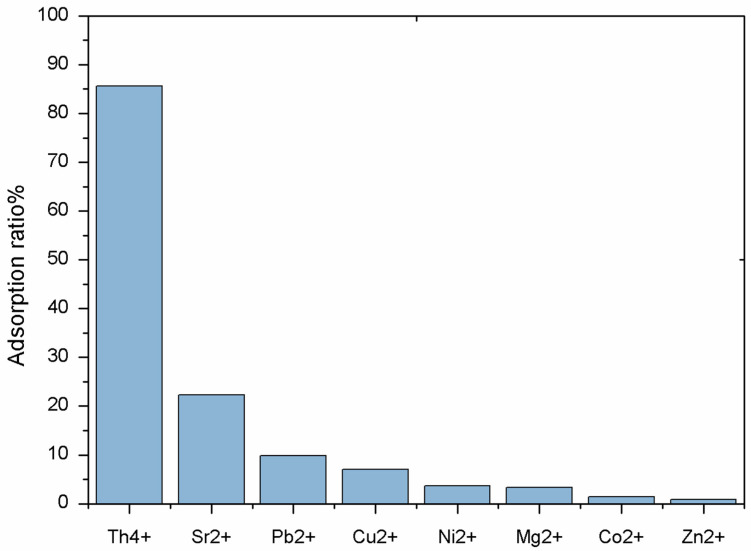
Selective adsorption of Th(IV) by AO-CELL. (Experiment conditions: solid/liquid ratio = 0.075 g/L; C_0_[Th(IV)] = 19.00 ± 0.10 mg/L; C_0_[M^2+^] = 8.0 × 10^−5^ mol/L; pH = 3.00 ± 0.05; T = 298.15 ± 1.00 K; t = 0.5 h).

As shown in Figure 8, under the set conditions, the adsorption percentage of Th(IV) by the AO-CELL can reach 85.64%, while the adsorption ratio% of the other metal ions is less than 10%, except for Sr^2+^, which is 22.3%. These results indicate that the AO-CELL has a high selectivity for Th(IV) and has a good potential for practical application. All the separation factor values are listed in Table 3.

#### 2.2.6. Effect of the Initial Concentration of Th(IV) and Adsorption Thermodynamics

**Figure 9:**
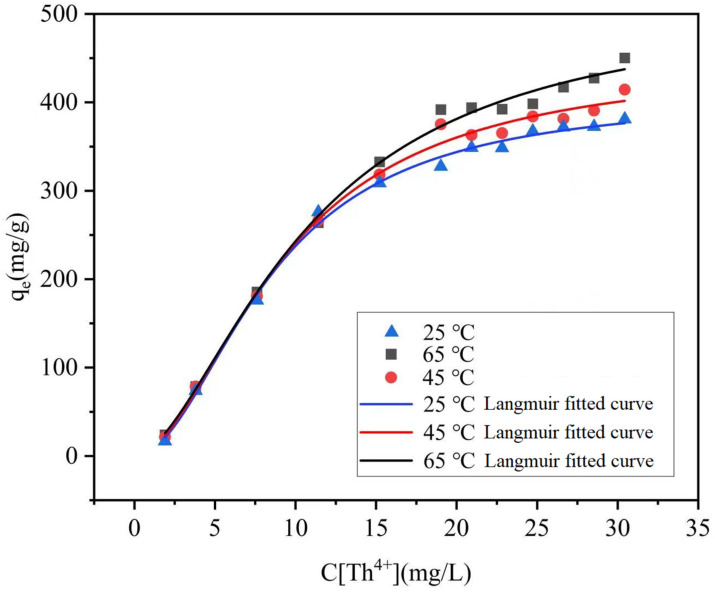
Effect of the initial concentration of Th(IV) and the temperature of the solution to the adsorption process. (Experiment conditions: temperature = 25.00 °C (9a)/45.00 °C (9b)/65.00 °C (9c); C_0_[Th(IV)] = 3.80~30.40 mg/L; solid/liquid ratio = 0.032 g/L; pH = 3.00 ± 0.05; t = 0.5 h).

The temperature of the solution system and the initial concentration of the adsorbed metal ions are important factors affecting the adsorption results. Figure 9 shows the relationship between the initial concentration of Th(IV) and the relative adsorption capacity of the AO-CELL at different temperatures (25 °C, 45 °C, 65 °C). Firstly, at the initial stage of the same temperature, the adsorption amount increases with the increasing of the initial concentration, and when the adsorption amount reaches a maximum value, it will not increase significantly. The maximum adsorption capacity of the AO-CELL for Th(IV) can reach 450 mg/g. Secondly, when the initial concentration is the same, the higher the temperature of the solution, the greater the adsorption capacity of the AO-CELL.

The study of the adsorption equilibrium will provide some information for judging the binding ability between the adsorption materials and the metal ions. The adsorption isotherms depend on specific parameters. The understanding of the parameters and the thermodynamic assumptions of these equilibrium models is helpful to understand the surface properties and the adsorption mechanism of the adsorbents [47].

Langmuir and Freundlich adsorption isotherm models are used in this paper. The assumptions of the Langmuir isotherm model are as follows: monolayer surface adsorption, adsorption sites are completely the same, and the adsorbed particles are completely independent. The linear form of the Langmuir equation is given by Equation (3)

C_e_/q_e_ = 1/(K_L_ · q_max_) + C_e_/q_max_, (3)

where C_e_ is the equilibrium concentration (mg/L) of Th(IV) in the solution, q_e_ is the adsorption capacity at the adsorption equilibrium (mg/g), q_max_ is the maximum adsorption capacity (mg/g), and K_L_ is the adsorption equilibrium constant (L/mg). Draw a straight line in a two-dimensional C_e_/q_e_ coordinate plot with slope and intercept equal to 1/q_max_ and 1/K_L_·q_max_, respectively.

K_L_ and q_max_ increased with increasing temperature. K_L_ increased with increasing temperature, indicating that the adsorption process was endothermic, and increasing the temperature could promote the interaction between Th(IV) and the active sites of the AO-CELL.

The Freundlich isotherm model is an empirical equation with no assumptions [48], and its linearization form is:

lnq_e_ = lnK_F_ + 1/n(lnC_e_) (4)

where K_F_ (mg^1-n^·L^n^·g^−1^) and n are characteristic constants related to the relative adsorption capacity and adsorption strength, respectively.

The Dubinin–Radushkevich (D-R) isotherm model was tested with the adsorption data to estimate the energy of adsorption to the nature of the adsorption process as physisorption or chemisorption. The linear form of this model (Equations (5)–(8)) is more general than the Langmuir model because it considers a heterogeneous surface [49].

Inq_e_ = Inq_max_ − K_DR_ε^2^ (5)

ε = RTln(1 + 1/C_e_) (6)

q_e_ = B_T_InA_T_ + B_T_InC_e_ (7)

E = 1/(2 K_DR_)^1/2^ (8)

where K_DR_, used to calculate the adsorption energy E (kJ·mol^−1^) by Equation (8), is the activity coefficient related to the mean adsorption energy, while ε is Polanyi potential, B_T_ (mg·g^−1^) is the heat of adsorption, and A_T_ (L·g^−1^) is the maximum binding energy between adsorbate and adsorbent. The calculated value of the adsorption mean free energy was found to be 11.18 kJ·mol^−1^ at 25 °C, and this result indicates a chemisorption adsorption process.

Compared with the Langmuir isotherm model, the theoretical adsorption capacity of the Freundlich isotherm model and the D-R model are quite different from the experimental adsorption capacity. The Langmuir isotherm model is more suitable than the Freundlich isotherm model to describe this adsorption process (the value of R^2^ ≈ 0.99). Correlation fitting results are collected in Table 4.

Thermodynamic analysis can help us to understand the adsorption process from a new perspective. In order to determine whether the reaction is spontaneous or not, one has to take into account variables such as energy and entropy. The experimental results show that the adsorption capacity (416–454–499 mg/g) also increases with the increasing of the temperature (25–45–65 °C). The van’t Hoff equation can be used to calculate the change of the corresponding thermodynamic parameters, including ΔG^0^ (kJ·mol^−1^), ΔH^0^ (kJ·mol^−1^), and ΔS^0^ (J·mol^−1^·K^−1^).

lnK_L_ = ΔS^0^/R − ΔH^0^/(R · T) (9)

ΔG^0^ = ΔH^0^ − TΔS^0^ (10)

where R is the ideal gas constant (8.314 J·mol^−1^·K^−1^) and T is the absolute temperature (K). The values of K_L_ at the different temperatures were used to calculate the thermodynamic parameters for the adsorption of Th(IV) ions on the AO-CELL. The detailed values of ΔG^0^, ΔH^0^, and ΔS^0^ are listed in Table 5.

When ΔG^0^ is negative, it indicates that the binding process of the AO-CELL with Th(IV) is spontaneous. With the increase of temperature, the ΔG^0^ value becomes smaller, indicating that the affinity of the AO-CELL increases with the increasing of the temperature, and the spontaneous binding trend of the AO-CELL with Th(IV) becomes more apparent [50]. It can also be seen from Table 4 that the ΔS^0^ value of the AO-CELL and the Th(IV) bonding process is greater than zero, indicating that the randomness of Th(IV) bonding with the adsorbed material interface increases. This result is related to the destruction of the Th(IV) hydration film before bonding with the AO-CELL [51]. ΔH^0^ is greater than zero, which also proves that the adsorption process is an endothermic reaction [52]. In conclusion, the adsorption process of Th(IV) in aqueous solution by the AO-CELL is a spontaneous endothermic reaction.

#### 2.2.7. Desorption and Reuse

**Figure 10:**
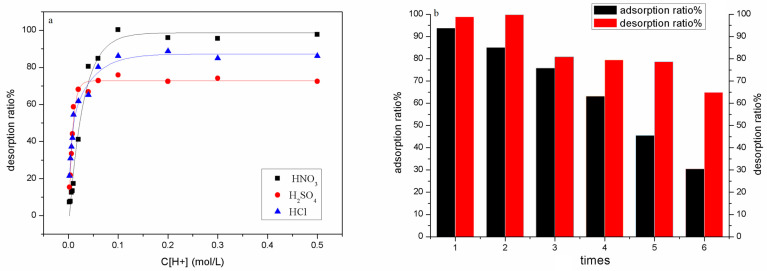
Desorption capacity and reuse capacity (**a**: Desorption capacity of AO-CELL under different acidic conditions; **b**: the adsorption regeneration experiment of AO-CELL. Reuse experiment conditions: solid/liquid ratio = 0.08 g/L; C_0_[Th(IV)] = 19.00 ± 0.10 mg/L; [H^+^] = 0.06 mol/L; T = 298.15 ± 1.00 K; t = 0.5 h).

The determination of the desorption capacity and the reuse capacity of the adsorbent is an important index to evaluate its practical application value [53], and it helps to understand the bonding mode between the metal ions and the adsorbents [43]. In this experiment, HCl (1.0 mol/L), H_2_SO_4_ (0.5 mol/L), and HNO_3_ (1.0 mol/L) were selected, respectively, to adjust the acidity of the solution. It can be seen from Figure 10a that when the acid concentration is very low, the desorption rate of thorium ion is also very low. The desorption ratio of the thorium ions increases rapidly with the increase of the acid concentration. However, the desorption percentage remains in a relatively fixed range after reaching a limit value. Compared with the three acids, nitric acid has the best desorption effect on the thorium ion, and the desorption percentage can reach 95% at 0.06 mol/L. In addition, the adsorption equilibrium between the thorium ion and the adsorbent tends to the desorption direction under strong acidic conditions, indicating that the combination between the thorium ion and the adsorbent may form ionic bonds or even coordination bonds through ion exchange [42]. As can be seen from the adsorption regeneration experiment results, as shown in Figure 10b, the AO-CELL can effectively repeat four cycles of the adsorption/desorption process for the same adsorbent (the adsorption ratio% > 60%). However, with the destruction of the adsorption material under acidic conditions, the adsorption capacity decreases significantly. Combined with desorption percentage, reuse experiment, and the low cost of the AO-CELL itself, etc., it can be explained that the amidoxime cellulose material has a good practical application value in the enrichment and separation of low-concentration thorium ions from wastewater solution.

## 3. Conclusions

In this paper, we synthetized and characterized amidoxime-functionalized cellulose, and used it as an adsorption material to separate Th(IV) ions from aqueous solution. Firstly, the composition and structure of the material were deduced by elemental analysis, SEM, EDS-mapping, XPS, and FT-IR. Secondly, the research results of the related influencing factors show that the adsorption reaction is a spontaneous exothermic process. The adsorption equilibrium movement is affected by the solid/liquid ratio of the adsorbent, the pH value of the solution, the solid/liquid contact time, and the initial concentration of Th(IV). Thirdly, the results of the study on the adsorption properties of the materials show that the adsorption capacity of the AO-CELL to Th(IV) can reach 500 mg/g under the optimal conditions. Due to the proper ionic radius and charge of Th(IV), it is easier to combine with the amidoxime group in the adsorbent in the form of chelate than the general low valence metal ion, so that the adsorbent has a good selectivity to the thorium ion. Under the premise of ensuring the adsorption capacity, the adsorbent material can be reused efficiently for four times. This provides experimental basis for studying its practical application value.

## 4. Materials and Methods

### 4.1. Materials

Cellulose (>98%, Xiyashiji Company, Linyi, China), acrylonitrile (AN, >99%, Xiyashiji Company, Chengdu, China), NH_2_OH·HCl (>98%, Xiyashiji Company, Linyi, China), methanol (>99%, Sinopharm Chemical Reagent Co., Ltd., Shanghai, China), isopropanol (>98%, Sinopharm Chemical Reagent Co., Ltd., China), Na_2_CO_3_ (>98%, Sinopharm Chemical Reagent Co., Ltd., Shanghai, China), sodium hydroxide (NaOH, >98%, Sinopharm Chemical Reagent Co., Ltd., Shanghai, China), phenol (C_6_H_6_O, >99%, Sinopharm Chemical Reagent Co., Ltd., Shanghai, China), potassium sodium tartrate tetrahydrate (C_4_H_4_O_6_KNa·4H_2_O, >99%, Xiyashiji Company, Linyi, China), HCl, H_2_SO_4_, and HNO_3_ (analytic reagent, Tianjin Guangfu Technology Development Co., Ltd., Shanghai, China), Arsenazo III (C_22_H_18_(As_2_)N_4_O_14_S_2_, >98%, Xiyashiji Company, Linyi, China), and *tetra*-methylammonium chloride ((CH_3_)_4_NCl, >98%, Sinopharm Chemical Reagent Co., Ltd., Shanghai, China), Th(NO_3_)_4_·6H_2_O, >98%, Xiyashiji Company, Linyi, China) were purchased as analytically pure without any further purification. All good quality analytical grade reagents were employed and the working solutions were prepared in ultra-pure water (Milli Q).

### 4.2. Characterization

The contents of N, C, H, and O were measured by an elemental analyzer (Vraio EL, Elementar, Hanau, German), which were used to estimate the conversion rate of the cyanidation and amidoxime reaction. The X-ray photoelectron spectroscopy (XPS) (Bruker BioSpin GmbH, Karlsruhe, Germany) was used to record the samples and to detect elements such as C, O, N, and Th(IV) in the AO-CELL. The morphological features of the cellulose and its derivatives were examined by the scanning electron microscope (SEM, Hitachi S-4800, Tokyo, Japan) and energy dispersive spectrometer (X-Max 80, Oxford Instruments, Oxford, UK). A Fourier transform infrared spectroscopy (FT-IR) (Nicolet Avatar 360, Thermo Nicolet, Waltham, MA, USA) spectrum spectrometer was used to characterize the material functional group identifications in the range between 4000 and 500 cm^−1^ by mixing 0.01 g of the material with 0.1 g KBr (spectroscopy grade) pellets. The concentration of Th(IV) was determined by spectrophotometer method (Perkin-Elmer, Waltham, MA, USA) at a wavelength of 652 nm using Th(IV)–Arsenazo(III) complex [17,54]. All the experimental data were the average of duplicate experiments, and the relative errors of data were less than 5%.

### 4.3. Batch Adsorption Experiments

The adsorption experiments of Th(IV) onto the AO-CELL were investigated by using the batch technique in 10 mL polyethylene tubes sealed with a screw-cap under certain conditions. Add the required reagents to the reaction tube, dilute with deionized water to approximately 5 mL, adjust the pH of the system with 0.2 mol/L nitric acid or sodium hydroxide, add the desired amount of adsorbent to the reaction system, and use deionized water to constant volume to 5.0 mL. After predetermined time intervals, the adsorbent was separated from the solutions by centrifugation. By measuring the concentration of thorium ion in the remaining solution, the adsorption rate% and adsorption capacity (q_e_ mg/g) under these conditions were determined.

The effects of the different factors on the adsorption were observed by changing the equilibrium time, ionic strength, initial adsorbent dose, pH value, and Th(IV) concentration. The amounts of adsorbed ions q_e_ (mg/g) and the percentage adsorption on the adsorbent (Adsorption ratio%) were calculated according to the following equations:

Adsorption ratio% = [(C_0_ − C_e_)/C_0_] ·100% (11)

q_e_ = [(C_0_ − C_e_)/m] · V (12)

where C_0_ (mg/L) and C_e_ (mg/L) are the Th(IV) concentrations of the liquid phase at initial time and equilibrium time, respectively; V is the volume of the solution (L); m is the weight of dry adsorbent used (g).

### 4.4. Research on the Effect of Different Factors

In the experiment studying the influence of the ionic strength on the adsorption process, different ionic strengths of solution were set by adding the calculated amount of Mg(NO_3_)_2_, Na_2_SO_4_, KNO_3_, and NaCl into the reaction system before constant volume. By measuring and calculating the adsorption ratio% of thorium ion adsorption by the adsorbent, the influence of different types and concentrations of anion and cation on the adsorption process was obtained. In the experiment studying the influence of the pH value on the adsorption process, the desired pH values were achieved by adding a negligible volume of 0.2 mol/L HNO_3_ or NaOH solutions.

In the study of the selective absorption of thorium ions by the AO-CELL, the Sr^2+^, Pb^2+^, Cu^2+^, Ni^2+^, Mg^2+^, Co^2+^, and Zn^2+^ were chosen as competing metal ions for Th(IV). The divalent metal ions concentration was measured by an atomic absorption spectrometer (AAnalyst 700, PerkinElmer Company, Waltham, MA, USA)

When studying the desorption capacity of Th(IV) by the AO-CELL, 0.2 mol/L HCl, 0.1 mol/L H_2_SO_4_, or 0.2 mol/L HNO_3_ were used to regulate the acidity of the solutions. While studying the reusability of the adsorbents, 0.06 mol/L HNO_3_ solution was used as the desorption system. The experimental process is as follows: under the same adsorption conditions, the AO-CELL that has completed the adsorption process is placed in a vacuum drying oven at 60 °C for 2 days. Different acid solutions were added into a 10.00 mL centrifuge tube to prepare a 5.00 mL solution system, and then the content of thorium in the solution was monitored. Desorption ratio is calculated by Equation (13).

Desorption ratio% = (C_d_ − C_ad_) · 100% (13)

C_d_ (mg·L^−1^) was the concentration of ions in the liquid supernatant after desorption by HNO_3_, and C_ad_ (mg·L^−1^) was the concentration of the ions adsorbed on the adsorbent during the adsorption experiment.

## Figures and Tables

**Table 1:** Elemental analysis of cellulose and its derivatives.

Samples	N(%)	C(%)	H(%)	O(%)
Cellulose	0	44.45	6.22	49.34
AN-CELL	5.89	49.68	6.11	38.32
AO-CELL	10.04	44.16	6.44	39.36

**Table 2:** Kinetic parameters for Th(IV) adsorbed onto AO-CELL.

Kinetic Model	Model Parameters	Results
pseudo-first order kinetic model	k_1_/(min^−1^)	2.31 × 10^−2^
	q_e1_/(mg/g)	16.29
	R^2^	0.3141
pseudo-second order kinetic model	k_2_/(g·mg^−1^·min^−1^)	4.82 × 10^−3^
	q_e2_/(mg/g)	294.12
	R^2^	0.9999
intraparticle diffusion model	k_int_/(mg·g^−1^·min^−0.5^)	2.291
	C/(mg/g)	273.81
	R^2^	0.7439

**Table 3:** The values of separation factor (S.F.) for Th^4+^ versus seven metal ions.

	Th^4+^/Sr^2+^	Th^4+^/Cu^2+^	Th^4+^/Mg^2+^	Th^4+^/Zn^2+^	Th^4+^/Pb^2+^	Th^4+^/Ni^2+^	Th^4+^/Co^2+^
S.F.	3.84	8.33	12.22	23.21	25.49	58.66	100.75

**Table 4:** The constants of Langmuir model, Freundlich model, and D-R model.

Langmuir model	**t/(°C)**	**K_L_ (L/g)**	**q_max_/(mg/g)**	**R_L_**	**R^2^**
	25.0	0.462	416.67	0.066–0.532	0.9873
	45.0	0.440	454.55	0.069–0.544	0.9931
	65.0	0.408	499.60	0.075–0.563	0.9849
Freundlich model	**t/(°C)**	**K_F_/(mg^1−n^L^n^g^−1^)**	**n**		**R^2^**
	25.0	139.60	2.6		0.7862
	45.0	177.24	3.2		0.8711
	65.0	180.06	3.0		0.8805
D-R model	**t/(°C)**	**K_DR_**	**q_max_/(mg/g)**	**E/(kJ/mol)**	**R^2^**
	25.0	4.00 × 10^−9^	48.58	11.18	0.7292
	45.0	5.00 × 10^−9^	18.98	10.00	0.7098
	65.0	4.00 × 10^−9^	16.30	11.18	0.7353

**Table 5:** Thermodynamic parameters.

Temperature	ΔG^0^/(kJ·mol^−1^)	ΔH^0^/(kJ·mol^−1^)	ΔS^0^/(J·mol^−1^·K^−1^)
25.0 °C	−34.60	5.86	135.71
45.0 °C	−37.32		
65.0 °C	−40.03		

## Data Availability

The data generated or analyzed during this study are available from the corresponding author on reasonable request.
